# Supplementary material for: Work ability during the COVID-19 pandemic: A cross-sectional study in a low-income urban setting in Brazil
Source: PLoS One. 2025 Jul 10;20(7):e0328127. doi: 10.1371/journal.pone.0328127 (PMC12244550; doi:10.1371/journal.pone.0328127)
Supplement: S1 File — (PDF) [file pone.0328127.s002.pdf]

Codebook for the database analyzed for the manuscript "Work ability during the COVID-19 pandemic: a cross-sectional study in a low-income urban setting in Brazil"

#	Variable	Description	Labels
1	**[part_id]**	Study participant identification number	
2	**[Sexo]**	Participant's gender	1=Female<br>2=Male
3	**[Age_group]**	Age categorized	1 = <45: Individuals who are younger than 45 years old.<br>2 = >=45: Individuals who are 45 years old or older.
4	**[RaceSkin_color_group]**	Race/color categorized	1=Black<br>2=White, mixed, other
5	**[Schooling_Group]**	Years of education	1=Up to 8 years of study<br>2=9 to 11 years of study<br>3=12 to 15 years of study<br>4=16 years or more of study
6	**[Marital_status_Group]**	Grouped marital status	1=Single<br>2=Married or living with a partner<br>3=Separated or widowed
7	**[Marital_status_Group2]**	Grouped marital status	1=Not married or living with a partner<br>2=Married or living with a partner
8	**[Young_apprentice]**	Self-reported working as a Young Apprentice	1=Yes 0=No
9	**[Body_mass_index]**	BMI classification	1=Underweight<br>2=Normal weight<br>3=Overweight<br>4=Obesity
10	**[Hypertension]**	Self-reported hypertension	1=Yes 0=No
11	**[Diabetes]**	Self-reported diabetes	1=Yes 0=No
12	**[Smoker]**	Self-reported smoker	1=Yes 0=No
13	**[COVID19_illness]**	Self-reported COVID-19	1=Yes 0=No
14	**[COVID19_illness_hosp]**	Self-reported hospitalization due to COVID-19	1=Yes 0=No
15	**[COVID19_vaccination]**	Self-reported vaccination against covid-19	1=Yes 0=No
16	**[Self_assessment_health]**	Self health assessment	1=Excellent or very good<br>2=Good or Moderate<br>3=Bad or very bad
17	**[Working]**	Self-reported if currently working or carrying out some financially remunerated activity	1=Yes 0=No
18	**[Number_of_works]**	Self-reported having more than one financially remunerated job or activity	0=Only one work/occupation<br>1=More than one work/occupation
19	**[Being_retired_work]**	Self-reported being retired and working	1=Yes 0=No
20	**[Bolsa_familia]**	Self-reported receiving the "Bolsa Família"	1=Yes 0=No
21	**[Pandemic_Emergency_Aid_2020]**	Self-reported receiving the COVID-19 emergency aid, 2020	1=Yes 0=No
22	**[Pandemic_Emergency_Aid_2021]**	Self-reported receiving the COVID-19 emergency aid, 2021	1=Yes 0=No
23	**[trab_pand]**	Same work as at the beginning of the pandemic	1=Yes 0=No
24	**[trab_perda]**	Lost your job or stopped carrying out any paid activity during the pandemic	1=Yes 0=No
25	**[sem_trab2___1]**	Self-referred reason for not working: Due to the pandemic (isolation, quarantine or social distancing)	1=Yes 0=No
26	**[sem_trab2___2]**	Self-referred reason for not working: Due to health problems or pregnancy	1=Yes 0=No
27	**[sem_trab2___3]**	Self-referred reason for not working: Just study	1=Yes 0=No
28	**[sem_trab2___4]**	Self-referred reason for not working: Don't want to work or are retired	1=Yes 0=No
29	**[sem_trab2___5]**	Self-referred reason for not working: No experience or qualifications	1=Yes 0=No
30	**[sem_trab2___6]**	Self-referred reason for not working: Won't find a job because he's too young or old	1=Yes 0=No
31	**[sem_trab2___7]**	Self-referred reason for not working: Can't find work in the region	1=Yes 0=No
32	**[sem_trab2___8]**	Self-referred reason for not working: You have to take care of household chores and/or family chores	1=Yes 0=No
33	**[sem_trab2___9]**	Self-referred reason for not working: Waiting for a response to get a job	1=Yes 0=No
34	**[sem_trab2___10]**	Self-referred reason for not working: Gave up looking for work	1=Yes 0=No
35	**[sem_trab2___11]**	Self-referred reason for not working: Another reason	1=Yes 0=No
36	**[Weekly_workload]**	Classification of weekly working hours	1=Up to 40 hours per week<br>2=More than 40 hours per week
37	**[Work_contract_status]**	Classification according to Work contract status	1=Informal Work<br>2=Formal Work
38	**[Work_from_home]**	The work or paid activity is outside the home	1=No, I only work from home. 0=Yes
39	**[ocup_1]**	Cleaning Sector = Domestic worker, day laborer, cook (in private households)	1=Yes 0=No
40	**[ocup_2]**	Cleaning = Cleaner, cleaning assistant, etc. (in public or private companies)	1=Yes 0=No
41	**[ocup_3]**	Administrative services = Office assistant, clerk	1=Yes 0=No
42	**[ocup_4]**	Administrative services = Secretary, receptionist	1=Yes 0=No
43	**[ocup_5]**	Commerce = Telemarketing operator	1=Yes 0=No
44	**[ocup_6]**	Commerce = Merchant (bar owner, shop owner, etc.)	1=Yes 0=No
45	**[ocup_7]**	Commerce = Shop clerk, store seller	1=Yes 0=No
46	**[ocup_8]**	Commerce = Door-to-door seller, sales representative, catalog seller (Avon, Natura, etc.)	1=Yes 0=No
47	**[ocup_9]**	Commerce = Street vendor (market seller, street vendor, kiosk vendor)	1=Yes 0=No
48	**[ocup_10]**	Food services = Cook and waiter (in restaurants, businesses)	1=Yes 0=No
49	**[ocup_11]**	Food services = Baker, butcher, pastry chef	1=Yes 0=No
50	**[ocup_14]**	Delivery and transportation = Driver (app driver, taxi driver, van driver, motorcycle taxi driver, bus driver)	1=Yes 0=No

Codebook for the database analyzed for the manuscript "Work ability during the COVID-19 pandemic: a cross-sectional study in a low-income urban setting in Brazil"

#	Variable	Description	Labels
**51**	**[ocup_16]**	Delivery and transportation = Motoboy	1=Yes 0=No
**52**	**[ocup_17]**	Delivery and transportation = Delivery person (restaurant, pharmacy, store, Uber Eats, IFood, Rappi, etc.)	1=Yes 0=No
**53**	**[ocup_19]**	Industry = Vehicle mechanic, industrial machine mechanic, etc.	1=Yes 0=No
**54**	**[ocup_20]**	Beauty and clothing = Artisan, seamstress, and shoemaker	1=Yes 0=No
**55**	**[ocup_21]**	Beauty and clothing = Hairdresser, manicurist, and similar professions	1=Yes 0=No
**56**	**[ocup_23]**	Industry = Production assistant, loading and unloading assistant	1=Yes 0=No
**57**	**[ocup_24]**	Education = Preschool teacher, elementary, middle, or high school teacher	1=Yes 0=No
**58**	**[ocup_25]**	Education = Pedagogue, language, music, art, and tutoring teacher	1=Yes 0=No
**59**	**[ocup_26]**	Health = Doctor, nurse, higher-level health professionals	1=Yes 0=No
**60**	**[ocup_27]**	Health = Technician, mid-level health professional	1=Yes 0=No
**61**	**[ocup_28]**	Health = Caregiver for children, sick, or elderly individuals	1=Yes 0=No
**62**	**[ocup_29]**	Security = Security guard, watchman, other protection service worker	1=Yes 0=No
**63**	**[ocup_31]**	Security = Doorman, janitor	1=Yes 0=No
**64**	**[ocup_32]**	Other sectors = Artist, religious leader (priest, pastor, etc.)	1=Yes 0=No
**65**	**[ocup_34]**	Other sectors = Other higher-level professions (lawyer, engineer, accountant, journalist, etc.)	1=Yes 0=No
**66**	**[ocup_35]**	Other sectors = Other mid-level technicians or professionals	1=Yes 0=No
**67**	**[ocup_36]**	Other sectors = Other	1=Yes 0=No
**68**	**[rm_pand]**	Self-reported whether there was a change in income during the pandemic	1=Yes 0=No
**69**	**[rm_pand2]**	If there was a change in income, did it increase or decrease	1=Decreased 2=Increased
**70**	**[Income_r$]**	Self-referred income in BRL	
**71**	**[Monthly_income]**	Monthly income	1=Up to USD 210<br>2=More than USD 210
**72**	**[risk_COVID19]**	Work-related risk of exposure to SARS-CoV-2	1=very high risk 2=high risk 3=medium risk 4=low risk
**73**	**[WAI]**	Work Ability Index	
**74**	**[WAI_bi]**	Work Ability Index - Dichotomized	1=inadequate work ability - WAI ranging from 7 to 36<br>2=adequate work ability - WAI ranging from 37 to 49
**75**	**[WAI_Group]**	Grouped Work Ability Index	1=Low (WAI 7-27)<br>2=Moderate (WAI 28-36)<br>3=Good (WAI 37-43)<br>4=Excellent (WAI 44-49)
