# Supplementary material for: Work ability during the COVID-19 pandemic: A cross-sectional study in a low-income urban setting in Brazil
Source: PLoS One. 2025 Jul 10;20(7):e0328127. doi: 10.1371/journal.pone.0328127 (PMC12244550; doi:10.1371/journal.pone.0328127)
Supplement: Attachment — Submitted filename: PONE-D-25-01676.pdf [file pone.0328127.s003.pdf]

PLOS ONE

Work ability during the COVID-19 pandemic: a cross-sectional study in a low-income urban setting in Brazil

--Manuscript Draft--

**Manuscript Number:**	PONE-D-25-01676
**Article Type:**	Research Article
**Full Title:**	Work ability during the COVID-19 pandemic: a cross-sectional study in a low-income urban setting in Brazil
**Short Title:**	Work ability during the COVID-19 pandemic in a low-income urban setting
**Corresponding Author:**	Guilherme S Ribeiro, M.D., M.Sc., Ph.D<br>Fundacao Oswaldo Cruz<br>Salvador, BA BRAZIL
**Keywords:**	Cross-sectional; Workers; Work Capacity Evaluation; Work Ability Index; COVID-19; SARS-CoV-2; Vulnerability; Low-income urban setting; Brazil
**Abstract:**	Work ability is a subjective concept that reflects the balance between an individual's perception of the physical, mental, and social demands of work and their competence and resources to meet those demands. The COVID-19 crisis significantly impacted health, work, and socioeconomic conditions worldwide. However, few studies have examined work ability in disadvantaged urban communities during this period. To analyze factors associated with work ability within the context of social vulnerability during the COVID-19 pandemic, we conducted a cross-sectional study in a low-income neighborhood in Salvador, Brazil, between February and June 2022. Sociodemographic, health, and labor data were collected, and work ability was assessed using the Work Ability Index (WAI), a widely used tool for evaluating work ability. Multivariable analyses based on a hierarchical model were run to investigate factors associated with low WAI scores. The study included 292 workers aged ≥16 years (59.6% women; median age 41 years). Most workers (84.6%) were classified as having adequate work ability based on their WAI scores. Multivariable analyses found that inadequate work ability was more frequent among women (prevalence ratio [PR]: 1.89, 95% confidence interval [CI]: 1.02-3.48), individuals who self-rated their health as moderate/good (PR: 5.91; 95% CI: 1.45-24.05) or poor/very poor (PR: 21.62; 95% CI: 5.14-90.91) compared to those with excellent/very good health, and those reporting diabetes (PR: 2.1; 95% CI: 1.13-3.9). Working >40 hours per week (PR: 0.47; 95% CI: 0.28-0.96) was negatively associated with inadequate work ability, suggesting that individuals with adequate work ability may be selected for longer working hours. A history of COVID-19 was not associated with inadequate work ability. These findings suggest that targeted interventions to improve work ability in low-income communities should prioritize women and workers with chronic health conditions, such as diabetes.
**Order of Authors:**	Ana Paula Cândido Oliveira
	Daniela Alencar Vieira
	Cristiane Wanderley Cardoso
	Tereza Magalhães
	Rosangela Oliveira Anjos
	Eduardo José Farias Borges Reis
	Kionna Oliveira Bernardes Santos
	Guilherme S Ribeiro, M.D., M.Sc., Ph.D
**Additional Information:**	
**Question**	**Response**
**Financial Disclosure**	Yes

Powered by Editorial Manager® and ProduXion Manager® from Aries Systems Corporation

Enter a financial disclosure statement that describes the sources of funding for the work included in this submission. Review the submission guidelines for detailed requirements. View published research articles from *PLOS ONE* for specific examples.<br><br>This statement is required for submission and **will appear in the published article** if the submission is accepted. Please make sure it is accurate.<br><br>**Funded studies**<br>Enter a statement with the following details:<br>• Initials of the authors who received each award<br>• Grant numbers awarded to each author<br>• The full name of each funder<br>• URL of each funder website<br>• Did the sponsors or funders play any role in the study design, data collection and analysis, decision to publish, or preparation of the manuscript?<br><br>Did you receive funding for this work?	
Please add funding details.<br>as follow-up to "**Financial Disclosure**<br><br>Enter a financial disclosure statement that describes the sources of funding for the work included in this submission. Review the submission guidelines for detailed requirements. View published research articles from *PLOS ONE* for specific examples.<br><br>This statement is required for submission and **will appear in the published article** if the submission is accepted. Please make sure it is accurate.	This study was supported by the Secretaria Municipal de Saúde de Salvador (Salvador Municipal Health Department), Brazil; the Fundação Oswaldo Cruz, Ministério da Saúde (Oswaldo Cruz Foundation, Ministry of Health) Brazil, and the Universidade Federal da Bahia (Federal University of Bahia), Brazil. The Conselho Nacional de Desenvolvimento Científico e Tecnológico (National Council for Scientific and Technological Development, CNPq) provided research scholarship to GSR (grant 311365/2021-3). The funders had no role in study design, data collection and analysis, decision to publish, or preparation of the manuscript.

Powered by Editorial Manager® and ProduXion Manager® from Aries Systems Corporation

**Funded studies**<br>Enter a statement with the following details:<br>• Initials of the authors who received each award<br>• Grant numbers awarded to each author<br>• The full name of each funder<br>• URL of each funder website<br>• Did the sponsors or funders play any role in the study design, data collection and analysis, decision to publish, or preparation of the manuscript?<br><br>Did you receive funding for this work?"	
Please select the country of your main research funder (please select carefully as in some cases this is used in fee calculation).<br>as follow-up to "**Financial Disclosure**<br><br>Enter a financial disclosure statement that describes the sources of funding for the work included in this submission. Review the submission guidelines for detailed requirements. View published research articles from *PLOS ONE* for specific examples.<br><br>This statement is required for submission and **will appear in the published article** if the submission is accepted. Please make sure it is accurate.<br><br>**Funded studies**<br>Enter a statement with the following details:<br>• Initials of the authors who received each award<br>• Grant numbers awarded to each author<br>• The full name of each funder<br>• URL of each funder website<br>• Did the sponsors or funders play any role in the study design, data collection and analysis, decision to publish, or preparation of the manuscript?<br><br>Did you receive funding for this work?"	BRAZIL - BR
**Competing Interests**	The authors have declared that no competing interests exist.

Use the instructions below to enter a competing interest statement for this submission. On behalf of all authors, disclose any competing interests that could be perceived to bias this work—acknowledging all financial support and any other relevant financial or non-financial competing interests.<br><br>This statement is required for submission and **will appear in the published article** if the submission is accepted. Please make sure it is accurate and that any funding sources listed in your Funding Information later in the submission form are also declared in your Financial Disclosure statement.<br><br>View published research articles from *PLOS ONE* for specific examples.<br><br>**NO authors have competing interests**<br><br>Enter: *The authors have declared that no competing interests exist.*<br><br>**Authors with competing interests**<br><br>Enter competing interest details beginning with this statement:<br><br>*I have read the journal's policy and the authors of this manuscript have the following competing interests: [insert competing interests here]*<br><br>* typeset	
**Ethics Statement**<br><br>Enter an ethics statement for this submission. This statement is required if the study involved:<br><br>• Human participants<br>• Human specimens or tissue<br>• Vertebrate animals or cephalopods<br>• Vertebrate embryos or tissues	All participants aged ≥18 years, as well as the legal guardians of a participant

Powered by Editorial Manager® and ProduXion Manager® from Aries Systems Corporation

• Field research<br><br>Write "N/A" if the submission does not require an ethics statement.<br><br>General guidance is provided below. Consult the submission guidelines for detailed instructions. **Make sure that all information entered here is included in the Methods section of the manuscript.**	

**Format for specific study types**<br><br>**Human Subject Research (involving human participants and/or tissue)**<br>• Give the name of the institutional review board or ethics committee that approved the study<br>• Include the approval number and/or a statement indicating approval of this research<br>• Indicate the form of consent obtained (written/oral) or the reason that consent was not obtained (e.g. the data were analyzed anonymously)<br><br>**Animal Research (involving vertebrate animals, embryos or tissues)**<br>• Provide the name of the Institutional Animal Care and Use Committee (IACUC) or other relevant ethics board that reviewed the study protocol, and indicate whether they approved this research or granted a formal waiver of ethical approval<br>• Include an approval number if one was obtained<br>• If the study involved *non-human primates*, add *additional details* about animal welfare and steps taken to ameliorate suffering<br>• If anesthesia, euthanasia, or any kind of animal sacrifice is part of the study, include briefly which substances and/or methods were applied<br><br>**Field Research**<br><br>Include the following details if this study involves the collection of plant, animal, or other materials from a natural setting:<br>• Field permit number<br>• Name of the institution or relevant body that granted permission	
**Data Availability**<br><br>Authors are required to make all data underlying the findings described fully available, without restriction, and from the time of publication. PLOS allows rare exceptions to address legal and ethical concerns. See the PLOS Data Policy and FAQ for detailed information.	Yes - all data are fully available without restriction

Powered by Editorial Manager® and ProduXion Manager® from Aries Systems Corporation

A Data Availability Statement describing where the data can be found is required at submission. Your answers to this question constitute the Data Availability Statement and **will be published in the article**, if accepted.<br><br>**Important:** Stating 'data available on request from the author' is not sufficient. If your data are only available upon request, select 'No' for the first question and explain your exceptional situation in the text box.<br><br>Do the authors confirm that all data underlying the findings described in their manuscript are fully available without restriction?	
**Describe where the data may be found in full sentences. If you are copying our sample text, replace any instances of XXX with the appropriate details.**<br><br>• If the data are **held or will be held in a public repository**, include URLs, accession numbers or DOIs. If this information will only be available after acceptance, indicate this by ticking the box below. For example: *All XXX files are available from the XXX database (accession number(s) XXX, XXX.).*<br>• If the data are all contained **within the manuscript and/or Supporting Information files**, enter the following: *All relevant data are within the manuscript and its Supporting Information files.*<br>• If neither of these applies but you are able to provide **details of access elsewhere**, with or without limitations, please do so. For example:<br><br>*Data cannot be shared publicly because of [XXX]. Data are available from the XXX Institutional Data Access / Ethics Committee (contact via XXX) for researchers who meet the criteria for access to confidential data.*<br><br>*The data underlying the results presented in the study are available from (include the name of the third party*	All relevant data are within the manuscript and its Supporting Information files.

and contact information or URL).<br>• This text is appropriate if the data are owned by a third party and authors do not have permission to share the data.<br><br>* typeset	
Additional data availability information:	

Manuscript
Click here to access/download;Manuscript;2025_01_16. Work ability_ in_ a_ low-income setting_Brazil.docx

Work ability during the COVID-19 pandemic: a cross-sectional study in a low-income urban setting in Brazil

Ana Paula Cândido de Oliveira[1,2], Daniela Alencar Vieira[1,2], Cristiane Wanderley Cardoso[1,2], Tereza Magalhães[1,3], Rosangela Oliveira dos Anjos[4], Eduardo José Farias Borges dos Reis[1], Kionna Oliveira Bernardes Santos[1], Guilherme Sousa Ribeiro[1,4] *

[1] Faculdade de Medicina, Universidade Federal da Bahia, Salvador, Brazil;
[2] Secretaria Municipal de Saúde de Salvador, Salvador, Brazil;
[3] Texas A&M University, College Station, United States of America;
[4] Instituto Gonçalo Moniz, Fundação Oswaldo Cruz, Salvador, Brazil.

*Corresponding author
E-mail: guilherme.ribeiro@fiocruz.br

Abstract

Work ability is a subjective concept that reflects the balance between an individual's perception of the physical, mental, and social demands of work and their competence and resources to meet those demands. The COVID-19 crisis significantly impacted health, work, and socioeconomic conditions worldwide. However, few studies have examined work ability in disadvantaged urban communities during this period. To analyze factors associated with work ability within the context of social vulnerability during the COVID-19 pandemic, we conducted a cross-sectional study in a low-income neighborhood in Salvador, Brazil, between February and June 2022. Sociodemographic, health, and labor data were collected, and work ability was assessed using the Work Ability Index (WAI), a widely used tool for evaluating work ability. Multivariable analyses based on a hierarchical model were run to investigate factors associated with low WAI scores. The study included 292 workers aged ≥16 years (59.6% women; median age 41 years). Most workers (84.6%) were classified as having adequate work ability based on their WAI scores. Multivariable analyses found that inadequate work ability was more frequent among women (prevalence ratio [PR]: 1.89, 95% confidence interval [CI]: 1.02-3.48), individuals who self-rated their health as moderate/good (PR: 5.91; 95% CI: 1.45-24.05) or poor/very poor (PR: 21.62; 95% CI: 5.14-90.91) compared to those with excellent/very good health, and those reporting diabetes (PR: 2.1; 95% CI: 1.13-3.9). Working >40 hours per week (PR: 0.47; 95% CI: 0.28-0.96) was negatively associated with inadequate work ability, suggesting that individuals with adequate work ability may be selected for longer working hours. A history of COVID-19 was not associated with inadequate work ability. These findings suggest that targeted interventions to improve work ability in low-income communities should prioritize women and workers with chronic health conditions, such as diabetes.

Keywords: Cross-sectional; Workers; Work Capacity Evaluation; Work Ability Index; COVID-19; SARS-CoV-2; Vulnerability; Low-income urban setting; Brazil

Introduction

The COVID-19 pandemic had both direct and indirect consequences worldwide. Beyond its health impacts, the pandemic and the measures taken to contain its spread exacerbated existing social vulnerabilities, such as poverty, unequal access to education, and precarious working conditions [1–3]. In the workforce, essential professionals, including those in health care, transportation, media, and food services, faced overwhelming demands and often increased working hours [2–5]. The mental strain from fears of contagion, heightened workloads, and staff shortages further contributed to an exhausting work environment, with significant consequences for mental health [2–7]. Sectors with a lower risk of direct exposure to contamination also had to adapt to the new realities, facing challenges such as the transition to remote work (often without adequate regulations), reduced paid working hours, employment contract suspensions, and widespread layoffs [3,8–12].

Across the world, several support and intervention policies were implemented to mitigate the impact of the labor market crisis caused by the pandemic [1,10]. In Brazil, the Emergency Employment and Income Maintenance Program played a crucial role in temporarily suspending employment contracts and reducing working hours with salary compensation [8]. These measures aimed to preserve jobs and income for formal workers while helping companies navigate the economic crisis without resorting to mass layoffs [13]. For the most socially vulnerable groups – comprising informal workers, individual microentrepreneurs, the self-employed, unemployed individuals, and families benefiting from Bolsa Familia, a federal income transfer program – an Emergency Aid subsidy was introduced to provide a minimum income [14].

Despite the importance of these social policies, their effectiveness varied widely, and they were unable to reach all those affected [15,16]. As a result, pre-existing social

vulnerabilities worsened, leading to increased poverty, unemployment, informal work, gender wage gap, and reduced access to education [1,5,10,17–19].

In this context, it was expected that the economic uncertainties and professional instability imposed by the COVID-19 pandemic would affect work ability globally, especially among the most vulnerable workers. Work ability is a multifaceted attribute that encompasses more than just technical or physical skills. It also considers an individual's adaptability to work demands and their ability to maintain satisfactory performance despite changes in health conditions or the work environment [20]. It is defined as a reflection of a worker's immediate and future well-being, closely linked to the physical, mental, and social demands of work activity [21–23]. Thus, work ability is a central element of both well-being and working life.

However, to our knowledge, no studies have examined work ability in low-income urban settings during the COVID-19 pandemic. In this study, we assessed the work ability of residents in an informal urban neighborhood in Brazil shortly after the peak of transmission of the Omicron BA.1 and BA.2 SARS-CoV-2 subvariants [24] and investigated factors associated with inadequate work ability.

Method

Study design and setting

This cross-sectional study was conducted from February 23 to June 22, 2022, in the Alto das Pombas neighborhood, an informal urban settlement in Salvador, Brazil. Alto das Pombas has a low-income population: 53% of residents registered in the Family Health Strategy (FHS) have 8 or fewer years of education, and 52% of the families earn up to the minimum Brazilian wage per month (US$ 254 as of June 2022) (unpublished data from the FHS, Secretary of Health of Salvador). The community is served by the governmental FHS, which provides free

preventive and primary health care centered on individuals and families in its territory. Each of the two FHS teams serving the community consists of one physician, one nurse, two nursing technicians, and six community health agents [25].

Participants were enrolled based on the following criteria: age ≥16 years, residency in the community (as defined by registration in the local FHS), and the ability to understand and respond to the survey questions. Although the Brazilian Apprenticeship Law [26] permits individuals as young as 14 years to work, we set the inclusion age limit at 16 years to avoid selecting young adolescents, whose likelihood of working was minimal. The study included both working and non-working individuals, enabling an examination of the employment context during the pandemic.

To obtain a representative sample from the neighborhood's population, we randomly selected 630 individuals from the 5,478 inhabitants registered in the community's FHS. As this study was part of a broader research project aimed at investigating various aspects of health and living conditions during the COVID-19 pandemic, the sample size was determined to provide approximately 4% precision for detecting events occurring in the population at a frequency of 50%, with a 95% confidence level. However, restrictions imposed by the pandemic limited the final sample size to 504 participants.

Data collection

The study team conducted home-visit interviews to collect sociodemographic, health, and occupational data using a mobile-based structured questionnaire on the Research Electronic Data Capture (REDCap®) software. If a participant could not be interviewed at home, the interview was conducted at the local FHS unit. Sociodemographic data collected included gender, age, marital status, education level, self-declaration of race/skin color, and income. Family-level data, such as enrollment in the Bolsa Familia program (a conditional cash transfer program provided by the federal government to families living in poverty) and in any

emergency aid programs established in 2020 and 2021 due to the COVID-19 pandemic, were also obtained.

Self-reported health data included weight, height, general health status (based on a five-item Likert scale to the question "*In general, how would you say your health is*?"), prior diagnoses of hypertension and diabetes, smoking history, and history of COVID-19. Occupational data included working status, type and number of job/occupations, weekly working hours, work as a young apprentice, working from home, retirement history, changes in income during the pandemic, level of work-related exposure to SARS-CoV-2 infection [27], loss or change of work since the beginning of the pandemic, and work ability. Work ability was assessed using the Brazilian Portuguese-translated and adapted version of the Work Ability Index (WAI) [23], which consists of ten questions across seven dimensions: 1) current work ability relative to the lifetime best, 2) work ability relative to job demands, 3) number of current diseases, 4) estimated work impairment due to diseases, 5) sick leave in the past year, 6) own prognosis of work ability in two years, and 7) mental resources [21]. The WAI was developed by Finnish researchers to evaluate how long individuals should continue working [28,29] and has been widely used to assess work ability [21,30–34]

Data analysis

Descriptive analyses were performed to characterize the study participants by work status (with or without occupation), using absolute and relative frequencies, as well as measures of central tendency and dispersion. Age was categorized into two groups (<45 and ≥45 years), based on the World Health Organization's definition of "aging worker", which considers individuals ≥45 years as potentially having reduced functional working capacities [20,35]. Individual monthly income was categorized into two groups: up to BRL 1,000 and more than BRL 1,000. This cutoff (approximately USD 210) was chosen to create two groups of roughly

equal size, using a rounded and meaningful BRL value. Work-related exposure risk to SARS-CoV-2 was classified into four levels: very high (health professionals performing aerosol-generating procedures), high (health professionals exposed to COVID-19 patients without performing such procedures), medium (professionals with close contact with potentially infected individuals), and low (professionals without significant contact with suspected or confirmed cases) [27].

The WAI score was calculated as the sum of points scored for each of the seven instrument dimensions initially categorized into four levels: low (7-27), moderate (28-36), good (37-43), and excellent (44-49 points) [22]. It was then recategorized into two levels: inadequate work ability (<37, covering the low and moderate categories, indicating the need to restore and improve work capacity) and adequate work ability (≥37, encompassing the good and excellent categories, indicating the importance of supporting and maintaining work ability), a classification also used in previous research [36–38].

To investigate factors associated with inadequate work ability, while accounting for the complex interrelationships between its determinants, we used a theoretical framework based on hierarchical relationships between the potentially explanatory variables and the dependent variable (inadequate work ability) [39]. This framework was developed using various multidimensional and integrated models of work ability [22]. It included three levels of related factors, considering both the hierarchical interrelationships among them and their connection to the dependent variable (Fig 1). In addition, demographic characteristics such as sex and age were included in the model for adjustment.

Fig 1. Theoretical framework for the hierarchical interrelationship of social, occupational, and health characteristics with work ability.

The most distal level included personal sociodemographic and economic social vulnerability indicators (e.g., race/skin color, education level, income, support from government income transfer programs – such as Bolsa Familia and Pandemic Emergency Aid – and marital status). These variables can potentially exert a direct influence on the other two levels of factors: the intermediate level, which includes occupational characteristics and work patterns (e.g., homeworking, number of working hours, employment status [formal vs informal], number of jobs, being a young apprentice, being retired and working, changes in employment and income during the pandemic); and the proximal level, which addresses health aspects (self-reported diabetes, high blood pressure, smoking, body mass index and prior COVID-19). Health aspects have been shown to significantly impact work ability [31]. Furthermore, specific questions on health status are included in the work ability construct (WAI), justifying this health block as the most proximal.

According to the theoretical framework model, bivariate and multivariable Poisson regression analyses with robust variance were used to identify factors associated with inadequate work ability. First, bivariate analyses were performed to estimate prevalence ratios (PR) and 95% confidence intervals (CI) and to select variables related to work ability at a significance level of 0.20 ($P < 0.20$) within each hierarchical level. Next, a series of multivariable analyses with backward elimination and adjustment for age and sex were run for each of the three blocks of variables (from the most distal to the most proximal level), including the within-block variables identified in the bivariate analyses. The first multivariable model included only the selected variables from the sociodemographic and economic block (distal level). Variables showing an association with work ability at a $P < 0.05$ were retained and included in the analyses of the intermediate and proximal levels, regardless of the P values obtained in these subsequent analyses. Similarly, selected variables from the occupational block (intermediate level) that showed an association with $P < 0.05$ were retained and included in the

health block (proximal level) analysis, regardless of the P values at this level. Variables associated with work ability at a P <0.05 in any of the blocks were defined as independently associated with work ability. Adjusted PR and 95% CI were calculated for the variables retained in each of the three blocks. The analyses were performed using IBM SPSS software version 20.0.0. The analyzed database and corresponding codebook are available in the supporting information files (S1 Database and S1 File).

Ethical aspects

All participants aged ≥18 years, as well as the legal guardians of a participant <18 years, signed an informed consent form. Participants <18 years old also signed an informed assent form. The study was approved by the Research Ethics Committee of the School of Medicine of Bahia, Federal University of Bahia (CAAE 55012022.5.0000.5577).

Results

Characteristics of the study participants

Of the 504 participants, 344 (68.3%) were female, and 470 (93.3%) self-reported as black or mixed race/skin color. The mean age was 45 years (standard deviation, SD = 17). Overall, education levels were low: 259 (51.4%) participants had not studied beyond 8 years of schooling. The majority (292; 57.9%) of participants were working, though a substantial portion (212; 42.1%) were not (Table 1). Compared to participants who were not working, those who were working were younger, included a higher proportion of men, were more likely to have a partner, and had more years of education (Table 1). A history of COVID-19 was more commonly reported among those who worked, with no participants reporting hospital admission due to the illness. In contrast, diabetes and hypertension were more prevalent among those who did not work. Among the 81 workers with at least one of these

two comorbidities, more than half (62; 76.5%) were aged 45 or older. Notably, 11 (61.1%) of the 18 workers with diabetes also had hypertension.

Table 1. Sociodemographic characteristics of the study population, according to occupational status, Salvador, Brazil, 2022

Characteristics	Total (N: 504)	Working (N: 292)	Not working (N: 212)
	Number (%)		
**Sex**			
Female	344 (68.3)	174 (59.6)	170 (80.2)
Male	160 (31.7)	118 (40.4)	42 (19.8)
**Age group**			
<45	257 (51.0)	167 (57.2)	90 (42.5)
≥45	247 (49.0)	125 (42.8)	122 (57.5)
**Race/Skin color**			
Black	282 (56.3)	172 (59.3)	110 (52.1)
White, mixed, other	219 (43.7)	118 (40.7)	101 (47.9)
**Marital status**			
Single	235 (46.6)	140 (47.9)	95 (44.8)
Married or living with a partner	216 (42.9)	137 (46.9)	79 (37.3)
Separated or widowed	53 (10.5)	15 (5.1)	38 (17.9)
**Education**			
Up to 8 years of study	110 (23.3)	48 (17.1)	62 (32.3)
9 to 11 years of study	119 (23.6)	58 (20.6)	61 (31.8)
12 to 15 years of study	220 (43.7)	156 (55.5)	64 (33.3)
16 years or more of study	24 (4.8)	19 (6.8)	5 (2.6)
**Body mass index**			
Underweight	23 (4.7)	9 (3.2)	14 (6.7)
Normal weight	155 (31.5)	89 (31.3)	66 (31.7)
Overweight	179 (36.4)	110 (38.7)	69 (33.2)
Obesity	135 (27.4)	76 (26.8)	59 (28.4)
**Self-reported clinical conditions**			
Smoker	37 (6.8)	20 (6.8)	17 (8.0)
Hypertension	160 (29.5)	74 (25.3)	86 (40.6)
Diabetes	51 (9.4)	18 (6.2)	33 (15.6)
**Self-reported COVID-19 and vaccination history**			
COVID-19 illness	166 (32.9)	104 (35.6)	62 (29.2)
COVID-19 vaccination (one or more doses)	497 (98.6)	290 (99.3)	207 (97.6)

The reasons for not being in paid work varied among the 212 participants who were not working. Of these, 63 (29.7%) were actively seeking employment, and 49 (23.1%) reported

being retired. Other reasons included health-related issues, including pregnancy (26; 12.2%), not wanting to work (22; 10.4%), dedication to studies (19; 9.1%), and household chores or caregiving for family members (16; 7.5%). Dedication to studies was primarily mentioned by men (17 of the 19), whereas household chores and caregiving were exclusively cited by women (16 of the 16). Social distancing due to the pandemic was reported as a reason by 5 (2.4%) participants. Additionally, 30 (14.2%) participants reported other reasons. The total exceeds 212 because some participants provided more than one reason for not working.

Additionally, 76 (26.0%) of the 292 participants who were currently working, reported changing occupations since the start of the pandemic. Among these 292 workers, 105 (36.0%) had formal employment (with a work contract and national social security registration), while 187 (64.0%) were in informal jobs (without a contract or social security registration). Most workers reported having only one work activity (236, 80.8%) and working up to 40 hours per week (193; 66.1%). Of the 276 workers who responded on their income, 119 (43.1%) earned less than BRL 1,000 (USD ~210) per month). The average monthly income was BRL 1,272.35, SD: 931.18, range: 100.00 – 6,000.00 (USD 267.34, SD: 194.64; range: 20.93 - 1,256.15).

The predominant employment sector was cleaning (59, 20.2%), followed by commerce (57, 19.5%), health (28, 9.6%), beauty and clothing (20, 6.8%), administrative services (16, 5.5%), delivery and transportation (15, 5.1%), security (15, 5.1%), construction (14, 4.8%), food services (11, 3.8%), education (8, 2.7%), and others (62, 21.2%); the sum of these numbers exceeds 292 because some participants had multiple work activities. Additionally, 41 (14.0%) participants reported working from home, primarily in food production, sales, beauty, education, and crafts; all of whom were women.

Work ability level

The mean WAI for participants who were working was 41.6 (SD: 5.6), with a median of 43. Adequate work ability (WAI ranging from 37 to 49) was observed in 247 (84.6%) workers, while inadequate work ability (WAI ranging from 7 to 36) was observed in 45 (15.4%) workers. Among those with adequate work ability, 119 (48.2%) had excellent and 128 (51.8%) had good work ability. Among those with inadequate work ability, 37 (82.2%) had moderate and 8 (17.8%) had low work ability.

Factors associated with inadequate work ability

Inadequate work ability was more common among women, individuals with a monthly income up to USD 210, recipients of the COVID-19 pandemic emergency aid, those working up to 40 hours per week, formally employed workers, individuals with low self-rated health, and those with a prior diagnosis of hypertension or diabetes mellitus (DM) ($P < 0.05$ for all variables; Table 2, in bold). Other variables not significantly associated with inadequate work ability, but included in the multivariable model, were age, enrollment in the Bolsa Familia program and self-reported prior COVID-19 illness ($P<0.20$; Table 2, in bold).

Table 2 – Frequency of inadequate work ability, according to sociodemographic, occupational, and health-related characteristics, Salvador-BA, 2022.

Characteristics	Categories	Number of participants	Number (%) with inadequate work ability	Crude PR (95% CI) [1]	P value [1]
**Sociodemographic**					
Sex	Female	174	33 (19.0)	1.87 (1.01 - 3.46)	**0.048**
	Male	118	12 (10.2)	1	
Age group	≥45 years	125	25 (20.0)	1.27 (0.70 - 2.31)	**0.063**
	<45 years	167	20 (12.0)	1	
Race/ ethnicity	Black	172	25 (14.5)	0.90 (0.52 – 1.56)	0.737
	White, mixed, other	118	19 (16.1)	1	
Marital status	Not married or living with a partner	155	23 (14.8)	0.92 (0.54 -1.58)	0.773
	Married or living with a partner	137	22 (16.1)	1	
Schooling	Up to 8 years of study	59	12 (20.3)	1.93 (0.47 – 7.87)	0.334
	9 to 11 years of study	58	5 (8.6)	0.82 (0.17 – 3.88)	
	12 to 15 years of study	156	26 (16.7)	1.58 (0.48 – 6.15)	
	16 years or more of study	19	2 (10.5)	1	
Monthly income	Up to USD 210	119	24 (20.2)	1.76 (1.00 – 3.09)	**0.049**
	More than USD 210	157	18 (11.5)	1	
Bolsa familia	Yes	32	8 (25.0)	1.76 (0.90 - 3.43)	**0.099**
	No	260	37 (14.2)	1	
COVID-19 emergency aid, 2020	Yes	150	25 (16.7)	1.16 (0.67 – 1.99)	0.594
	No	139	20 (14.4)	1	
COVID-19 emergency aid, 2021	Yes	104	22 (21.2)	1.73 (1.01 - 2.95)	**0.044**
	No	188	23 (12.2)	1	
**Occupational**					
Young apprentice	Yes	6	0	NA	NA
	No	286	45 (15.7)		
Number of works	Only one work/occupation	236	35 (14.8)	0.83 (0.44 – 1.57)	0.569
	More than one work/occupation	56	10 (17.9)	1	
Work contract status	Formal work	105	10 (9.5)	1	**0.045**

	Informal work	187	35 (18.7)	1.97 (1.01 - 3.81)	
Weekly workload	Up to 40 hours per week	193	36 (18.7)	1	**0.026**
	More than 40 hours per week	98	8 (8.2)	0.44 (0.21 – 0.91)	
Work from home	Yes	41	10 (24.4)	1.74 (0.94 - 3.24)	**0.079**
	No	250	35 (14.0)	1	
Being retired and working	Yes	12	3 (25.0)	1	0.326
	No	280	42 (15.0)	0.60 (0.22 – 1.66)	
Work-related risk of exposure to SARS-CoV-2 [2]	Very high or high risk	13	1 (7.7)	0.43 (0.06 – 2.94)	0.574
	Medium risk	184	27 (14.7)	0.81 (0.47 – 1.41)	
	Low risk	94	17 (18.1)	1	
Income changes during the pandemic	No change	100	12 (12.0)	0.77 (0.33 – 1.83)	0.463
	Decreased	145	26 (17.9)	1.15 (0.54 – 2.48)	
	Increased	45	7 (15.6)	1	
Same work as before the pandemic	Yes	208	34 (16.3)	1	0.491
	No	84	11 (13.1)	0.80 (0.43 – 1.51)	
**Health-related**					
Self-assessment of health	Excellent or very good	79	2 (2.5)	1	**<0.001**
	Good or Moderate	197	32 (16.2)	6.42 (1.58 - 26.14)	
	Bad or very bad	16	11 (68.8)	27.16 (6.65-110.96)	
Body mass index range	Low and normal weight	98	18 (18.4)	1.31 (0.76 – 2.28)	0.330
	Overweight and obesity	186	26 (14.0)	1	
Smoking	Yes	20	1 (5.0)	1	0.233
	No	272	44 (16.2)	3.24 (0.47 -22.78)	
Hypertension	Yes	74	19 (25.7)	2.15 (1.27 - 3.66)	**0.005**
	No	218	26 (11.9)	1	
Diabetes	Yes	18	8 (44.4)	3.29 (1.81 - 5.98)	**<0.001**
	No	274	37 (13.5)	1	
Self-reported covid-19 illness	Yes	104	20 (19.2)	1.45 (0.85 - 2.47)	**0.178**
	No	188	25 (13.3)	1	

NOTE: NA, not applicable as it was not possible to estimate.

[1] Bivariate Poisson regression with robust variance was used to estimate prevalence ratios (PR), 95% confidence intervals (CI) and P values.

[2] The work-related risk of exposure to SARS-CoV-2 was defined in the methods section.

The first multivariable model using the sociodemographic data (model A, Table 3), showed a higher prevalence of inadequate work ability among women compared to men (PR: 1.89, 95% CI: 1.02–3.48) and among workers aged ≥45 years compared to those <45 years (PR: 1.69; 95% CI: 0.99–2.89). The second multivariable model, which added occupational variables to the variables selected in model A (model B, Table 3), found that those working more than 40 hours per week were less likely to have inadequate work ability than those working up to 40 hours per week (RP: 0.47; 95% CI: 0.28 – 0.96). The third multivariable model (model C, Table 3), which incorporated health-related variables along with those selected in model B, found higher prevalences of inadequate work ability among those who self-rated their health as good/moderate (PR: 5.91; 95% CI 1.45 – 24.05) or bad/very bad (RP: 21.62; 95% CI: 5.14 – 90.91), compared to those who self-rated their health as excellent/very good, and among those with diabetes (PR: 2.10; 95% CI: 1.13 – 3.90). Notably, the effect of sex on the occurrence of inadequate work ability was reduced after incorporating the occupational and, in particular, health-related variables (models B and C, Table 3), indicating that the effect of sex on work ability is at least partially mediated by these factors. The Akaike Information Criterion (AIC) for each model showed that model C, which included sociodemographic, occupational, and health-related variables, provided the best fit (Table 3).

Table 3. Multivariable hierarchical model analyses of factors associated with inadequate work ability among workers in the community of Alto das Pombas, Salvador, 2022

Characteristic	Model A. Sociodemographic variables	Model B. Sociodemographic and occupational variables	Model C. Sociodemographic, occupational, and health-related variables
	Adjusted PR (95% CI) and model AIC		
**AIC**	257.502	251.788	229.395
**Sociodemographic**			
Sex			
Male	1	1	1
Female	1.89 (1.02 - 3.48)	1.75 (0.95 - 3.23)	1.27 (0.70 - 2.31)
Age			
<45 Years	1	1	1
≥45 Years	1.69 (0.99 - 2.89)	1.61 (0.94 - 2.76)	1.52 (0.90 – 2.58)
**Occupational characteristic**			
Weekly workload			
Up to 40 hours per week		1	1
More than 40 hours per week		0.47 (0.28 – 0.96)	0.53 (0.26 – 1.08)
**Health-related**			
Self-assessment of health			
Excellent or very good			1
Good or moderate			5.91 (1.45 - 24.05)
Bad or very bad			21.62 (5.14 - 90.91)
Diabetes			
No			1
Yes			2.10 (1.13 - 3.90)

Discussion

Our study, conducted in a low-income urban community in Salvador, Brazil, two years after the onset of the COVID-19 pandemic, found that 57.9% of residents aged 16 and older were working, with about a quarter having changed their work during the pandemic. The proportion of working individuals in our study was similar to the occupation rate (including both paid and unpaid work, the latter in support of a family member's work) in the working-age population in Brazil and Salvador during the same study period (56.8% and 54.7%, respectively) [40].

However, the working participants differed from those who were not working in terms of sociodemographic and health-related characteristics. Women, individuals aged ≥45 years, those with fewer years of education, and those with a higher frequency of comorbidities were less likely to engage in paid work. These differences may reflect gender disparities, labor market discrimination, and cultural factors that overwhelm women with domestic duties and assign them caregiving roles in families. As a result, women often face challenges in balancing paid work with these essential but time-consuming responsibilities. In addition, these disparities highlight significant barriers for older individuals, those with less education, and those with chronic health conditions in securing and maintaining employment.

We also found that most (84.6%) of the study participants who were working had adequate working ability, with mean and median WAI scores similar to those reported in studies from several countries, such as Iran, Brazil, Italy, and Poland, which included workers from various sectors [41–44]. However, none of these studies were conducted in socially vulnerable populations. Therefore, the similarity between the WAI from our study and those from other settings may suggest that, despite the high level of social deprivation in our population, their work ability was likely not affected. Alternatively, the economic crisis induced by the COVID-19 pandemic may have acted as a filter, retaining individuals with better work ability in the

workforce, while those with lower work ability may have been more vulnerable to job loss. Furthermore, the economic crisis may have introduced a positive bias in the assessment of work ability, as those who were able to maintain their jobs may have experienced greater job satisfaction, influencing their WAI scores. Hence, it is important to consider whether the high frequency of adequate work ability is, at least in part, due to a "healthy worker effect", where those who remain working tend to be healthier and better able to work. Future studies examining work ability should take the socioeconomic context into account when interpreting the WAI.

In general, higher WAI scores are expected in younger people, while lower scores are typically observed in older individuals [20,23,35,36,45–47]. Although our study did not find a significant association between age and work ability, we observed a trend in the expected direction. After adjusting for sex, the prevalence ratio of inadequate work ability for those aged ≥45 years compared to those <45 years was 1.69, with a 95% CI ranging from 0.99 and 2.89. This suggests that such an association may exist in the population from which our sample was drawn. However, the sample size may not have been large enough to conclusively demonstrate this association, in contrast to another study conducted during the pandemic that did find such an association [48].

Regarding gender's role in work ability, women had an 89% higher frequency of inadequate work ability compared to men. However, after adjusting for occupational and health-related characteristics, this effect was reduced to 27% and lost statistical significance, suggesting that the gender differences in work ability are primarily due to gender-related disparities in occupational and, mainly, health conditions, rather than any inherent gender trait. Previous studies have similarly found higher prevalences of inadequate work ability among women, attributing this to a combination of family responsibilities, physical and mental stress associated with extended work hours, and the multiple roles women often fulfill [34,49–53]. Our hierarchical analysis supports that workload and lower self-assessed health status mediate

the effect of gender on work ability. Additionally, in our sample, all domestic tasks and unpaid family caregiving were performed solely by women, underscoring the persistent asymmetry in the gender-based division of unpaid activities [54]. Despite social progress in recent decades, many women in our society continue to be culturally and socially conditioned to take on the caregiver and household roles [55], leaving them with less time for outside work.

We also found that workers earning up to BRL 1,000 (USD 210) per month had a significantly higher prevalence of inadequate work ability (PR: 1.76). Although the PR remained relatively stable after adjusting for age and sex during the selection of variables from the first block (PR: 1.68, data not shown), statistical significance was lost and the variable was not retained in the model. However, the existence of an association between income and work ability cannot be ruled out, as a bidirectional relationship between these factors is plausible. Prospective studies should explore this connection, as lower earnings may increase the risk of engaging in work activities that impair work ability, while inadequate work ability may, in turn, lead to lower earnings—potentially creating a vicious cycle. The non-significant but positive association of inadequate work ability with both being a recipient of Bolsa Família and the 2021 COVID-19 emergency aid further supports a relationship between income and work ability, as beneficiaries of these cash transfer programs typically have lower incomes.

In the bivariate analysis of occupational variables, informal workers – who do not contribute to the national social security program and lack legal rights to vacation, paid sick leave, and an end-of-year bonus – had a significantly higher prevalence of inadequate work ability than formal workers. However, this association did not hold in the multivariable analyses. The link between precarious working conditions and lower work ability had been observed before the pandemic [34]. In addition, a separate study that tracked work ability over 12 months during the pandemic, focusing on a sample predominantly composed of public servants with job stability and remote work arrangements, found that work ability remained

largely stable. This finding suggests that more favorable working conditions may positively affect work ability [42]. Future studies should further explore the impact of employment insecurity, lack of rights, and limited protection on work ability.

The analysis of other occupational factors revealed an unexpected association: workers with a regimen of over 40 hours per week were less likely to experience inadequate work ability than those working 40 hours or fewer. This association, which persisted after adjusting for age and sex, contradicts previous studies that suggest excessive workload negatively impacts work capacity through effects like fatigue and stress [41,47]. A likely explanation for our finding is the presence of the "healthy worker effect", where workers with adequate work ability may be more inclined toward and/or selected for longer work hours. In other words, work capacity may have influenced workload rather than the reverse. Further research is needed to clarify the complex relationships between work regimen characteristics, including contract type (formal vs. informal), and work ability across various occupational contexts.

Regarding the health-related variables, it is well established that chronic diseases can significantly impact work capacity [20,22,31]. Although hypertension was initially associated with inadequate work ability, this relationship did not remain significant in the multivariable model, likely due to high correlations with other health-related variables such as prior diabetes diagnosis and self-rated health status. Notably, having diabetes and self-rating health as less than excellent or very good were the factors most strongly associated with inadequate work ability. This aligns with findings from previous research, which show that individuals with poor self-rated health had a sixfold increase in the prevalence of limited work ability compared with those who perceived themselves to be in good health [22]. Given the high prevalence of hypertension and diabetes in our study population [25,56] and in other working groups [22,45,57], promoting a lifestyle that includes healthy eating, physical activity, and the proper diagnosis and management of these conditions is essential to support work capacity.

The finding that self-assessed health status was strongly associated with inadequate work ability reinforces using self-perceived health as a key indicator of work capacity, as suggested by other studies [58]. Furthermore, it underscores the importance of considering both objective clinical aspects and subjective health perceptions when evaluating work capacity and developing strategies to enhance workers' well-being and productivity.

We found no association between self-reported COVID-19 and work ability. In our study, virtually every participant had been vaccinated against COVID-19, and none of the self-reported COVID-19 cases required hospitalization, suggesting they experienced mild illness. Additionally, the WAI has primarily been used to assess the effect of chronic illness on work ability [31,41,59], and the impact of acute mild illness on its score remains less clear. Similar to our findings, another Brazilian study following 1,211 workers found no differences in work ability between individuals infected and not infected with SARS-CoV-2, both at baseline (June to September 2020) and during follow-up (completed in October 2021) [42]. In contrast, a small study of 33 workers hospitalized for COVID-19 in Italy found a decline in work ability, with pre-COVID-19 WAI scores measured at hospital discharge dropping from good to medium levels one month later (mean WAI: 41.9 and 35.6, respectively) [60].

This study has several limitations. First, due to its cross-sectional design, we were unable to establish causality for the observed associations. As mentioned earlier, reverse associations may have occurred, such as the relationship between workload and work ability. Second, the WAI instrument was originally designed to assess retirement eligibility related to aging and disability in the workforce [23,61], and it was not used for this purpose in our study. Moreover, since no specific cut-off points for the WAI have been validated for Brazilian workers [31], we relied on the cut-off points established by the instrument's developers. However, alternative cut-off points for younger populations have been proposed [62]. We also acknowledge that the WAI instrument is not comprehensive, as it does not account for lifestyle

factors such as alcohol consumption and physical activity, which are essential components of a multidimensional approach to evaluating work ability.

Third, we did not address relevant contextual factors related to work, such as working conditions, working environment, prior professional experience, or the above-mentioned lifestyle factors, all of which have been shown to influence work ability, both before and during the pandemic [44,46–48]. Additionally, data on COVID-19 history were collected via self-report, which may have led to misclassification, as some participants may have mistakenly identified other respiratory or febrile illnesses as COVID-19. Conversely, using a laboratory-confirmed diagnosis could have resulted in an underreporting of cases, given the limited availability of diagnostic tests, especially during the early months of the pandemic when testing was prioritized for severe cases. We also did not explore the relationship between post-COVID-19 symptoms or long COVID-19 and work ability, preventing a more comprehensive analysis of the effects of COVID-19 and work capacity.

Furthermore, the pandemic context may have influenced participants' perceptions of their work ability, as maintaining paid work during the socioeconomic crisis could have led to a more positive self-assessment of their overall capacity to work. Lastly, the relatively small sample size limited our power to detect weaker associations as significant and resulted in wide 95% confidence intervals for some prevalence ratios, indicating low precision in their measurement. Nevertheless, our study provides valuable exploratory insights into potential factors contributing to inadequate work ability within the context of urban vulnerability and a global health crisis.

Conclusions

In summary, we found that nearly 15% of the workers from a low-income urban community in Brazil had inadequate work ability. This study was conducted amid the social

and economic crisis caused by the COVID-19 pandemic, which may have influenced the profile of the working population and their capacity to work. The multidimensional hierarchical model used to explore associations between sociodemographic, occupational, and health-related characteristics with inadequate work ability revealed that these three dimensions can significantly impact work capacity. Notably, health-related characteristics were identified as the most significant contributors. This finding highlights the importance of policies and practices that prioritize health promotion, inequities, vulnerable people, access to adequate health care, and proper management of medical conditions to enhance work capacity.

Acknowledgments

We thank all the residents of Alto da Pombas for their support for the researchers in conducting the study, especially those who generously agreed to participate. We also extend our thanks to the community health workers and other members of the local Family Health Program, students from the Universidade Federal da Bahia, and the technical staff from Fundação Oswaldo Cruz, who assisted the research team with field activities, data collection, and data management.

References

1. UNRR, UNU. Rethinking risks in times of COVID-19. Reliefweb. 2022 [cited 19 May 2022]. Available: https://reliefweb.int/report/world/rethinking-risks-times-covid-19
2. Sim MR. The COVID-19 pandemic: major risks to healthcare and other workers on the front line. Occup Environ Med. 2020;77: 281–282. doi:10.1136/oemed-2020-106567
3. Burdorf A, Porru F, Rugulies R. The covid-19 pandemic: One year later – an occupational perspective. Scand J Work Environ Heal. 2021;47: 245–247. doi:10.5271/sjweh.3956
4. Fiho JMJ, Assunção AÁ, Algranti E, Garcia EG, Saito CA, Maeno M. A saúde do trabalhador e o enfrentamento da COVID-19. Rev Bras Saúde Ocup. 2020;45: 10–12. doi:10.1590/2317-6369ed0000120
5. Santos KOB, Fernandes R de CP, Almeida MMC de, Miranda SS, Mise YF, Lima MAG

de. Trabalho, saúde e vulnerabilidade na pandemia de COVID-19. Cad Saude Publica. 2020;36: 1–14. doi:10.1590/0102-311X00178320

6. Burdorf A, Porru F, Rugulies R. The COVID-19 (Coronavirus) pandemic: consequences for occupational health. Scand J Work Environ Health. 2020;46: 229–230. doi:10.5271/sjweh.3893

7. Machado MH, Militão JB, Machado AV. Os trabalhadores invisiveis da saúde – a invisibilidade em questão. In: Valda F, Viegas P, Duraes M, Gosch C, Cosac AS, editors. A Pandemia e o Trabalho em Saúde: vozes do cotidiano. Editora Rede Unida; 2022. p. 6. doi:10.18310/9788554329464

8. Souza D de O. As dimensões da precarização do trabalho em face da pandemia de Covid-19. Trab Educ e Saúde. 2021;19. doi:10.1590/1981-7746-sol00311

9. Praun L. A Espiral da Destruição: legado neoliberal, pandemia e precarização do trabalho. Trab Educ e Saúde. 2020;18: 1981–7746. doi:10.1590/1981-7746-sol00297

10. International Labour Office. Global Wage Report 2020–21: Wages and minimum wages in the time of COVID-19. Geneva; 2020.

11. Burdorf A, Porru F, Rugulies R. The COVID-19 (Coronavirus) pandemic: Consequences for occupational health. Scand J Work Environ Heal. 2020;46: 229–230. doi:10.5271/sjweh.3893

12. Belzunegui-Eraso A, Erro-Garcés A. Teleworking in the Context of the Covid-19 Crisis. Sustainability. 2020;12: 3662. doi:10.3390/su12093662

13. BRASIL. Governo lança Programa Emergencial de Manutenção do Emprego para enfrentar efeitos econômicos da Covid-19. In: Ministério da Economia [Internet]. 2020 [cited 5 Jul 2023]. Available: https://www.gov.br/economia/pt-br/assuntos/noticias/2020/abril/governo-lanca-programa-emergencial-de-manutencao-do-emprego-para-enfrentar-efeitos-economicos-da-covid-19

14. Bourdeau W, Silva IC, Oliveira MLC de F. Nota Técnica No. 15. Rede Pesqui Solidária. 2020;15: 8p. Available: https://redepesquisasolidaria.org/wp-content/uploads/2020/07/boletimpps_15_10julho.pdf

15. Meireles T, Menezes V, Simoni S, Flores P, Lazzari E, Guicheney H, et al. Nota Técnica No. 14. Rede Pesqui Solidária. 2020;14: 13p. Available: https://redepesquisasolidaria.org/wp-content/uploads/2020/07/boletimpps_14_3julho.pdf

16. Reis AC, Silva EP, Meirelles CM. O novo normal no campo da educação: da aparência à essência. Princípios. 2020;1: 225–245. doi:10.4322/principios.2675-6609.2020.160.010

17. Barreto ML, Aquino EML. Pandemia de Covid-19: reflexões sobre seus impactos, incertezas e controvérsias. In: Buss PM, Burger P, editors. Diplomacia da saúde: respostas globais à pandemia. Rio de Janeiro: Fiocruz; 2021. pp. 33–43. Available: www.insp.mx

18. Palhares G, Milanezi J, Santos JM dos, Simões L, Brandão R. Nota Técnica No. 7. Rede Pesqui Solidária. 2020; 1–21. Available: https://redepesquisasolidaria.org/wp-content/uploads/2020/05/boletim-7-pps.pdf

19. Granemann S. Crise econômica e a Covid-19: rebatimentos na vida (e morte) da classe trabalhadora brasileira. Trab Educ e Saúde. 2021;19: 1–12. doi:10.1590/1981-7746-sol00305

20. WHO (World Health Organizacion). Aging and working capacity: report of a WHO study group. World Health Organization. Geneva: (WHO technical report series: 835).; 1993.

21. Ilmarinen J. The Work Ability Index (WAI). Occup Med (Chic Ill). 2006;57: 160–160. doi:10.1093/occmed/kqm008

22. Gould R, Ilmarinen J, Järvisalo J, Koskinen S. Dimensions of work ability. Results from the Health 2000 Survey. Gould R, Ilmarinen J, Järvisalo J, Koskinen S, editors. International Archives of Occupational and Environmental Health. Helsinki (Finland): Finnish Centre for Pensions; 2008. Available: https://www.julkari.fi/bitstream/handle/10024/78055/dimensions_of_work_ability_7.pdf

23. Tuomi K, Ilmarinen J, Jahkola A, Katajarinne L, Tulkki A. Índice de Capacidade para o Trabalho. Traduzido por Frida Marina Fischer (coord.). 1a. ed. (1. São Carlos: Edufscar; 2010.

24. Aguilar Ticona JP, Xiao M, Li D, Nery N, Hitchings M, Belitardo EMMA, et al. Extensive transmission of SARS-CoV-2 BQ.1* variant in a population with high levels of hybrid immunity: A prevalence survey. Int J Infect Dis. 2024;139: 159–167. doi:10.1016/j.ijid.2023.11.039

25. Oliveira APC de, Vieira DA, Jesus AA de. Unidade de Saúde da Família. In: Araújo FB, Mota JC, Mucci MB, Jacobina RR, Reis EJFB dos, editors. Entre olhares e vivências no Alto das Pombas Entre olhares e vivências no Alto das Pombas: educação em saúde em um bairro popular. Salvador,BA: EdUFBA; 2020. pp. 277–298. Available: http://repositorio.ufba.br/ri/handle/ri/33623

26. BRASIL. Law n. 10,097 - Brazilian Apprenticeship Law. 10097 Dec 19, 2000. Available: https://www.planalto.gov.br/ccivil_03/leis/l10097.htm

27. Bahia G do E. NOTA TÉCNICA COE - SAÚDE Nº 53 DE 06 DE ABRIL DE 2020. 2020.

28. Ilmarinen J, Tuomi K, Eskelinen L, Nygård CH, Huuhtanen P, Klockars M. Summary and recommendations of a project involving cross-sectional and follow-up studies on the aging worker in Finnish municipal occupations (1981-1985). Scand J Work Environ Health. 1991;17 Suppl 1: 135–41. Available: http://www.ncbi.nlm.nih.gov/pubmed/1792527

29. Tuomi K, Ilmarinen J, Klockars M, Nygård CH, Seitsamo J, Huuhtanen P, et al. Finnish research project on aging workers in 1981-1992. Scand J Work Environ Health. 1997;23 Suppl 1: 7–11. Available: http://www.ncbi.nlm.nih.gov/pubmed/9247990

30. Cadiz DM, Brady G, Rineer JR, Truxillo DM. A Review and Synthesis of the Work Ability Literature. Wang M, editor. Work Aging Retire. 2019;5: 114–138. doi:10.1093/workar/way010

31. Martinez MC, Latorre M do RD de O, Fischer FM. Capacidade para o trabalho: revisão de literatura. Cien Saude Colet. 2010;15: 1553–1561. doi:10.1590/S1413-

81232010000700067

32. Truxillo DM, Cadiz DM, Brady GM. COVID-19 and its implications for research on work ability. Wang M, editor. Work Aging Retire. 2020;6: 242–245. doi:10.1093/WORKAR/WAAA016

33. van den Berg TIJ, Elders LAM, de Zwart BCH, Burdorf A. The effects of work-related and individual factors on the Work Ability Index: a systematic review. Occup Environ Med. 2009;66: 211–220. doi:10.1136/oem.2008.039883

34. Cordeiro TMSC e, Araújo TM de. Capacidade para o trabalho entre trabalhadores do Brasil. Rev Bras Med do Trab. 2016;14: 262–274. doi:10.5327/Z1679-443520165115

35. Ilmarinen J. Aging workers. Occup Environ Med. 2001;58: 546–552. doi:10.1136/oem.58.8.546

36. Fischer F, Borges N, Rotenberg L, Latorre M, Soares NS, Rosa P, et al. A (in) capacidade para o trabalho em trabalhadores de enfermagem. Rev Bras Med Trab Belo Horiz. 2005;3: 97–103.

37. Milosevic M, Golubic R, Knezevic B, Golubic K, Bubas M, Mustajbegovic J. Work ability as a major determinant of clinical nurses' quality of life. J Clin Nurs. 2011;20: 2931–2938. doi:10.1111/j.1365-2702.2011.03703.x

38. Yong M, Nasterlack M, Pluto R-P, Elmerich K, Karl D, Knauth P. Is health, measured by work ability index, affected by 12-hour rotating shift schedules? Chronobiol Int. 2010;27: 1135–1148. doi:10.3109/07420528.2010.490111

39. Victora CG, Huttly SR, Fuchs SC, Olinto MT. The role of conceptual frameworks in epidemiological analysis: a hierarchical approach. Int J Epidemiol. 1997;26: 224–227. doi:10.1093/ije/26.1.224

40. IBGE. PNAD Contínua. In: Pequisa Nacional Por Amostra de Domicílios - Contínua [Internet]. 2021 [cited 2 Jul 2023]. Available: https://painel.ibge.gov.br/pnadc/

41. Amirmahani M, Hasheminejad N, Tahernejad S, Reza Tohidi Nik H, Nik HRT, M A, et al. Evaluation of work ability index and its association with job stress and musculoskeletal disorders among midwives during the Covid-19 pandemic. Med del Lav. 2022;113: 1–11. doi:10.23749/mdl.v113i4.12834

42. Andrade MA, Castro CSMM, Batistão M V., Mininel VA, Sato TO. Occupational Profile, Psychosocial Aspects, and Work Ability of Brazilian Workers During COVID-19 Pandemic: IMPPAC Cohort. Saf Health Work. 2022;13: 104–111. doi:10.1016/j.shaw.2021.11.004

43. Barbic F, Minonzio M, Cairo B, Shiffer D, Zamuner AR, Cavalieri S, et al. Work ability assessment and its relationship with cardiovascular autonomic profile in postural orthostatic tachycardia syndrome. Int J Environ Res Public Health. 2020;17: 1–15. doi:10.3390/ijerph17217836

44. Grabara M, Sadowska-Krępa E. Occupational and leisure time physical activity of territorial army soldiers during the COVID-19 pandemic in the context of their perceived work ability. Int J Occup Med Environ Health. 2022;35: 327–337. doi:10.13075/ijomeh.1896.01881

45. Hunter JR, Brett A, Cripps A, Supiah HT, I GM, Vicendês D, et al. Machine Translated

by Google e Saúde Pública Relações entre Atividade Física , Capacidade para o Trabalho , Absenteísmo e Presenteísmo na Austrália e Nova Zelândia Adultos durante o COVID-19 Machine Translated by Google. 2021.

46. Koziel RJ, Friedrich JC, Rudolph CW, Zacher H, Balducci C, Cadiz D. Age-Differentiated Leadership and Healthy Aging at Work: Evidence from the Early Stages of the COVID-19 Pandemic. Int J Environ Res Public Health. 2021;18: 12509. doi:10.3390/ijerph182312509

47. Lu Y, Liu Q, Yan H, Gao S, Liu T. Job burnout and its impact on work ability in biosafety laboratory staff during the COVID-19 epidemic in Xinjiang. BMC Psychiatry. 2021;21: 543. doi:10.1186/s12888-021-03555-x

48. Hunter JR, Meiring RM, Cripps A, Suppiah HT, Vicendese D, Kingsley MI, et al. Relationships between Physical Activity, Work Ability, Absenteeism and Presenteeism in Australian and New Zealand Adults during COVID-19. Int J Environ Res Public Health. 2021;18: 12563. doi:10.3390/ijerph182312563

49. Bellusci SM, Fischer FM. Envelhecimento funcional e condições de trabalho em servidores forenses. Rev Saude Publica. 1999;33: 602–609. doi:10.1590/S0034-89101999000600012

50. Linhares JE. Avaliação da capacidade para o trabalho: análise frente ao envelhecimento funcional de servidores públicos em um município da região sul. 2017. Available: http://repositorio.utfpr.edu.br/jspui/handle/1/2352

51. Vasconcelos SP, Fischer FM, Reis AOA, Moreno CR de C. Fatores associados à capacidade para o trabalho e percepção de fadiga em trabalhadores de enfermagem da Amazônia ocidental. Rev Bras Epidemiol. 2011;14: 688–697. doi:10.1590/S1415-790X2011000400015

52. Costa CSN da, Freitas EG de, Mendonça LC de S, Alem MER, Coury HJCG. Capacidade para o trabalho e qualidade de vida de trabalhadores industriais. Cienc e Saude Coletiva. 2012;17: 1635–1642. doi:10.1590/S1413-81232012000600026

53. Oliveira Júnior P. Índice de capacidade para o trabalho (ICT): uma avaliação da capacidade laboral dos profissionais de enfermagem portadores de doenças crônicas não transmissíveis. Universidade Federal de Uberlândia. 2018. doi:10.14393/ufu.di.2018.940

54. Garcia BC, Marcondes GDS. As desigualdades da reprodução: homens e mulheres no trabalho doméstico não remunerado. Rev Bras Estud Popul. 2022;39: 1–23. doi:10.20947/S0102-3098a0204

55. Pinheiro L, Medeiros M, Costa J, Barbosa A de H. Gênero é o que importa : determinantes do trabalho doméstico não remunerado no Brasi. Texto para Discussão, n 2920. 2023; 44. doi:http://dx.doi.org/10.38116/td2920

56. Araujo F, Silva KC, Reis EJFB, Oliveira APC de, Anjos RO dos, Silva VF. Perfil epidemiológico de uma comunidade coberta pela Estratégia de Saúde da Família em Salvador-BA. APS em Rev. 2022;4: 208–214. doi:10.14295/aps.v4i3.206

57. Ilmarinen J. Towards a longer worklife! Ageing and the quality of worklife in the European Union. Ageing and the quality of worklife in the European …. 2008. Available: http://www.stm.fi/Resource.phx/eng/publc/special/worklife.htx.i122.pdf

58. Eskelinen L, Kohvakka A, Merisalo T, Hurri H, Wägar G. Relationship between the self-assessment and clinical assessment of health status and work ability. Scand J Work Environ Health. 1991;17 Suppl 1: 40–7. Available: http://www.ncbi.nlm.nih.gov/pubmed/1838820

59. Oliveira Júnior PC de, Mendes PC, Jesus EHA de, Santos F de O. AVALIAÇÃO DO ÍNDICE DE CAPACIDADE PARA O TRABALHO (ICT) DOS PROFISSIONAIS DE ENFERMAGEM PORTADORES DE DOENÇAS CRÔNICAS NÃO TRANSMISSÍVEIS. Hygeia - Rev Bras Geogr Médica e da Saúde. 2021;17: 227–242. doi:10.14393/Hygeia17063158

60. Barbic F, Minonzio M, Rigo S, Shiffer D, Putti G, Rinaldi L, et al. Work ability in Post-acute COVID-19 syndrome: one-month follow-up after Hospital discharge. Saf Health Work. 2022;13: S222–S223. doi:10.1016/j.shaw.2021.12.1446

61. Milani D. (Dissertação) Capacidade para o trabalho, sintomas osteomusculares e qualidade de vida entre operadores de máquinas agrícolas. Universidade Federal de São Carlos- UFSCar. 2011.

62. Kujala V, Remes J, Ek E, Tammelin T, Laitinen J. Classification of Work Ability Index among young employees. Occup Med (Chic Ill). 2005;55: 399–401. doi:10.1093/occmed/kqi075

Supporting information

S1 Database: Excel database containing the data analyzed for the manuscript "*Work ability during the COVID-19 pandemic: a cross-sectional study in a low-income urban setting in Brazil*".

S1 File: Codebook for the database analyzed for the manuscript *"Work ability during the COVID-19 pandemic: a cross-sectional study in a low-income urban setting in Brazil"*.

Fig 1

Click here to access/download;Figure;Fig1.TIF

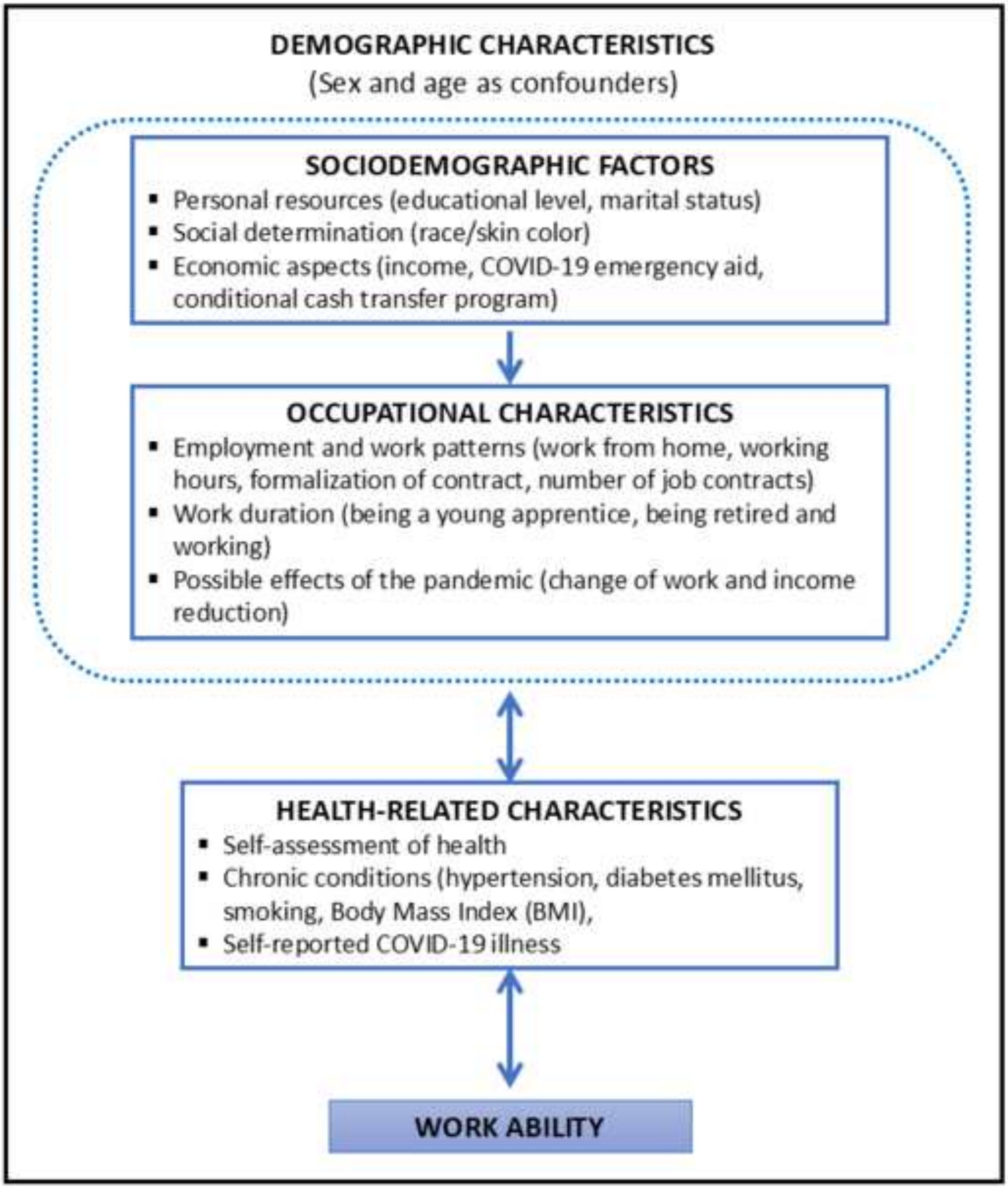

S1 Database

Click here to access/download
Supporting Information
S1 Database.xls

Click here to access/download
Supporting Information
S1 File_ Database codebook.pdf
